# Genome sequencing and analysis of *Talaromyces pinophilus* provide insights into biotechnological applications

**DOI:** 10.1038/s41598-017-00567-0

**Published:** 2017-03-28

**Authors:** Cheng-Xi Li, Shuai Zhao, Ting Zhang, Liang Xian, Lu-Sheng Liao, Jun-Liang Liu, Jia-Xun Feng

**Affiliations:** State Key Laboratory for Conservation and Utilization of Subtropical Agro-bioresources, College of Life Science and Technology, Guangxi University, 100 Daxue Road, Nanning, Guangxi 530004 People’s Republic of China

## Abstract

Species from the genus *Talaromyces* produce useful biomass-degrading enzymes and secondary metabolites. However, these enzymes and secondary metabolites are still poorly understood and have not been explored in depth because of a lack of comprehensive genetic information. Here, we report a 36.51-megabase genome assembly of *Talaromyces pinophilus* strain 1–95, with coverage of nine scaffolds of eight chromosomes with telomeric repeats at their ends and circular mitochondrial DNA. In total, 13,472 protein-coding genes were predicted. Of these, 803 were annotated to encode enzymes that act on carbohydrates, including 39 cellulose-degrading and 24 starch-degrading enzymes. In addition, 68 secondary metabolism gene clusters were identified, mainly including T1 polyketide synthase genes and nonribosomal peptide synthase genes. Comparative genomic analyses revealed that *T. pinophilus* 1–95 harbors more biomass-degrading enzymes and secondary metabolites than other related filamentous fungi. The prediction of the *T. pinophilus* 1–95 secretome indicated that approximately 50% of the biomass-degrading enzymes are secreted into the extracellular environment. These results expanded our genetic knowledge of the biomass-degrading enzyme system of *T*. *pinophilus* and its biosynthesis of secondary metabolites, facilitating the cultivation of *T*. *pinophilus* for high production of useful products.

## Introduction

*Talaromyces pinophilus*, formerly designated *Penicillium pinophilum*, is a fungus that produces biomass-degrading enzymes such as α-amylase^1^, cellulase^2^, endoglucanase^3^, xylanase^2^, laccase^4^ and α-galactosidase^2^. In one study, a blended enzyme cocktail produced by *T. pinophilus* and *Chrysoporthe cubensis* improved the efficiency of hydrolysis of glucan and xylan in sugarcane bagasse for glucose and xylose production, compared with enzymes from a single strain^2^. A relatively high level of β-glucosidase activity is observed in *T*. *pinophilus* under solid state fermentation^5^. Therefore, *T. pinophilus* is considered a potential alternative to *Trichoderma reesei* for cellulase production and efficient biomass hydrolysis. *T. pinophilus* produces a variety of medically useful metabolites such as 3-*O*-methylfunicone, which is used to inhibit mesothelioma cell motility^6^, and talaromycolides 1–3, 5 and 11, which inhibit the growth of the human pathogen methicillin-resistant *Staphylococcus aureus* ^7^.

The fungal strain *T. pinophilus* 1–95 was isolated from the soil of a dried, ploughed field in Wuzhou, China. This strain produces a highly efficient, calcium-independent α-amylase^1^. Application of calcium-independent α-amylase in starch conversion avoids problems caused by addition of calcium ions^8^. Additionally, we found that *T. pinophilus* 1–95 produces 1.21 ± 0.30 U/mL of filter-paper cellulase, 10.72 ± 0.74 U/mL of carboxymethylcellulose cellulase, 0.71 ± 0.02 U/mL of *p*-nitrophenyl-β-cellobioside cellulase, 0.27 ± 0.01 U/mL of *p*-nitrophenyl-β-glucopyranoside cellulase and 41.93 ± 2.84 U/mL of xylanase activities in submerged flask cultivation (data not shown). However, a comprehensive understanding of the biomass-degrading enzyme system in this fungus is still lacking.

We describe the *de novo* whole-genome assembly of *T. pinophilus* strain 1–95, a nearly complete genome sequence of a high biomass-degrading enzyme-producing species in the genus *Talaromyces*. Carbohydrate-active enzyme (CAZyme) genes and secondary metabolism gene clusters were observed in the sequenced genome. Comparative genomic analysis suggested that *T*. *pinophilus* harbors more biomass-degrading enzymes and secondary metabolites than other related filamentous fungi. In addition, the predicted secretory protein patterns of *T. pinophilus* 1–95 were analyzed.

## Results

### Genome sequencing, assembly and annotation

**Figure 1:**
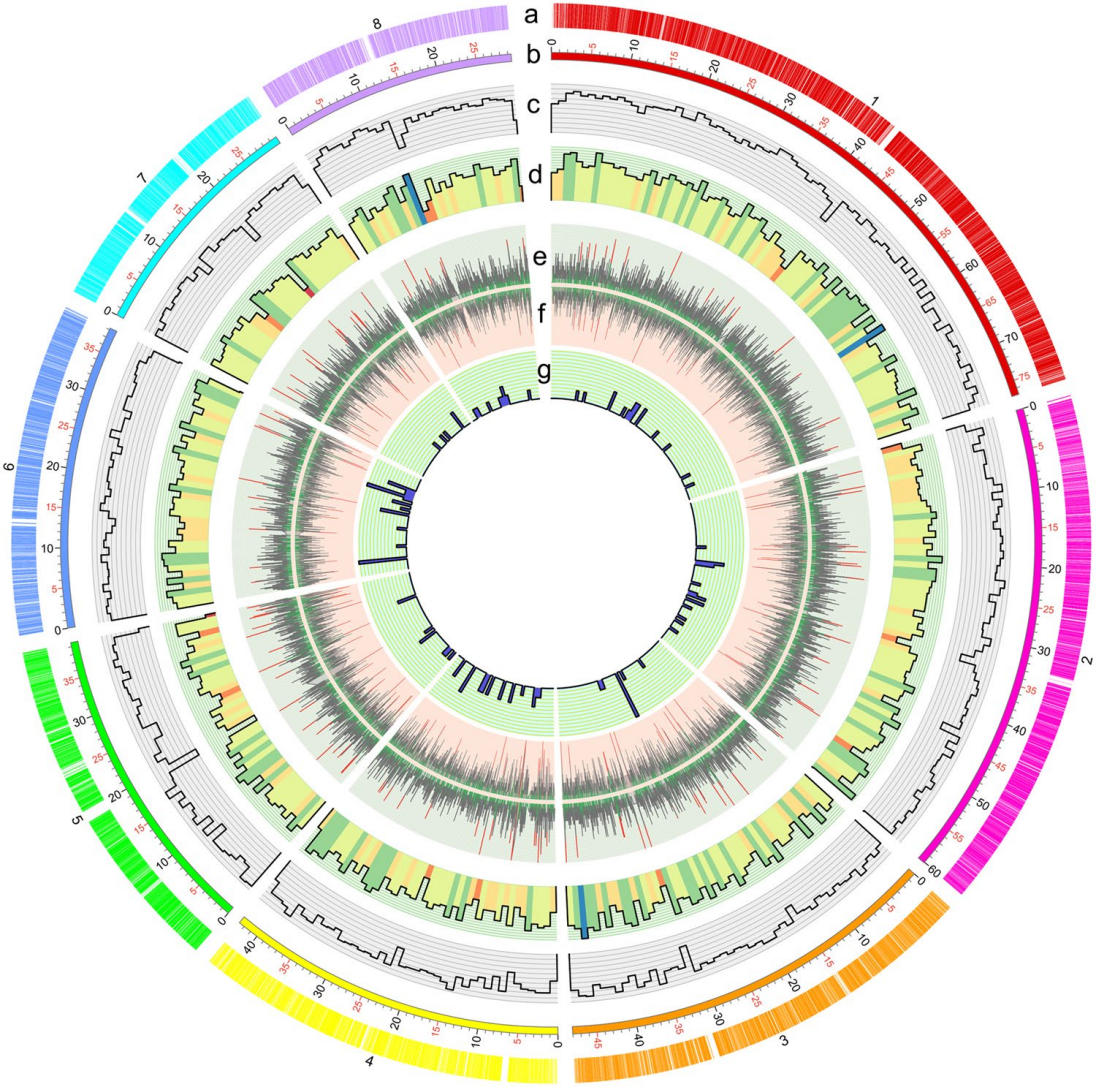
Circular map of genomic features of the *T. pinophilus* 1–95 genome. (**a**) Location of predicted genes. Numbers represent predicted chromosomes. (**b**) Schematic representation of genomic characteristics of *T. pinophilus* pseudogenome (Mb scale). (**c**) Gene density represented by number of genes in 100-kb nonoverlapping windows. (**d**) Gene density of genes annotated by the GO database in each 100-kb window. (**e**) Exon positions of protein-coding genes (circle). Red, exon number (>10) in a gene; green, one exon included. (**f**) Intron positions of protein-coding genes (circle). Red, exon number (>10) in a gene; green, no intron included. (**g**) The tRNA density represented by number of tRNAs in 100-kb nonoverlapping windows.

Genome sequencing of the fungal strain *T. pinophilus* 1–95 (CGMCC No. 2645), isolated from soil in a dried, ploughed field in Wuzhou, China, was performed using a combination of single molecule real-time (SMRT) DNA sequencing and next generation sequencing technology. A high-quality genome sequence of *T. pinophilus* 1–95 was generated on the PacBio RS II platform. Approximately 1.94 Gb of clean subreads, with sequences from a single pass of a polymerase on a single strand of an insert within a SMRTbell template and no adapter sequences with an N50 size of 10,045 bp and average length 8,102 bp were generated. Additionally, a paired-end (PE) library with a 500-bp average insert size was constructed using the Illumina HiSeq 4000 platform, and 3.88 Gb clean, short-sequence PE reads were generated with a length of 125 bp. Reads were used to correct wrong bases in the assembled genome sequence on the PacBio RS II platform. Finally, a 36.51-Mb genome of *T. pinophilus* 1–95 was generated with 159-fold coverage. This size was in accordance with the estimated genome size of 28–36 Mb for three *Talaromyces* species^9–11^. The genome was covered by nine scaffolds, including eight large scaffolds (accession number CP017344-CP017351 in GenBank) without gaps (Fig. 1) and a smaller circular scaffold (accession number CP017352 in GenBank). The N50 and N90 sizes of the scaffolds were, respectively, 4.80 Mb and 2.99 Mb (Table 1). Telomeric repeats (primarily 5ʹ-TTAGGG-3′) were found at both ends of all large scaffolds except for scaffold number 2, for which a telomeric repeat was only found at one end, possibly due to incompleteness of the scaffold sequence data. We inferred that the *T. pinophilus* 1–95 genome consisted of eight chromosomes (Fig. 1). Additionally, a smaller circular scaffold of 31.73 kb was assembled as a mitochondrial genome (Table 1). Several sequenced *Penicillium* and *Aspergillus* species such as *P*. *oxalicum* 114-2^12^ and *Aspergillus niger* ATCC 1015^13^ are also predicted to have eight chromosomes. The overall GC content of the *T. pinophilus* 1–95 genome was 46.23%. The GC content was 50.08% for the coding sequences and 24.84% for the mitochondrial genome. Other general features of the *T. pinophilus* 1–95 genome are in Table 1.

**Table 1:** Genome features of *Talaromyces pinophilus* 1–95.

Genome features	Value
**Nuclear genome**	
Size of assembled genome (Mbp)	36.51
GC content of assembled genome (%)	46.25
N50 Length (bp)	4,804,168
N90 Length (bp)	2,993,891
Maximum length (bp)	7,684,667
Minimum length (bp)	2,941,929
All protein-coding genes	13,472
Protein-coding genes (≥60 aa)	13,450
Average gene length (bp)	1,602.97
Average number of introns per gene	2.07
Average intron length (bp)	75.36
Average exons per gene	3.07
Average exon length (bp)	470.18
tRNA genes	107
**Mitochondrial genome**	
Size (bp)	31,729
GC content (%)	24.84
tRNA genes	25

In total, 13,472 protein-coding genes were predicted from the genome assembly using five *ab initio* gene prediction programs: Augustus (http://bioinf.uni-greifswald.de/augustus/), GeneMark-ES (http://exon.gatech.edu/GeneMark/), Genewise (http://www.ebi.ac.uk/Tools/psa/genewise/), SNAP^14^ and an unsupervised learning system program Glean version 1 (https://sourceforge.net/projects/glean-gene/). The number of coding genes was significantly higher than other filamentous fungi that produce biomass-degrading enzymes (see Supplementary Table S1). Of the *T. pinophilus* coding genes, 8162 (60.58%), were annotated in the Gene Ontology (GO) database (http://geneontology.org/), 12,828 (95.21%) in the UniProt database (http://www.uniprot.org/), 12,946 (96.09%) in the NCBI non-redundant (NR) protein sequences database (ftp://ftp.ncbi.nlm.nih.gov/blast/db/) and 4437 (32.93%) in the Clusters of Orthologous Groups of proteins database (http://www.ncbi.nlm.nih.gov/COG/). A total of 6817 (50.6%) genes belonging to 331 pathways were also annotated in the Kyoto Encyclopedia of Genes and Genomes (KEGG) database (http://www.kegg.jp/) (see Supplementary Fig. S1). The coding regions of the predicted genes constituted almost 53.37% of the genome, with an average length of 1446.5 bp. BUSCO^15^ and CEGMA^16^ were used to evaluate the integrity of the genome assembly and prediction of gene sets. A BUSCO set for fungi comprising 1438 single-copy ortholog genes from more than 100 fungal strains was used to evaluate the genome assembly and gene sets of *T. pinophilus* 1–95. More than 98% of the orthologous genes matched with genes in the genome and gene sets of *T. pinophilus* 1–95 (see Supplementary Table S2). Using CEGMA, 238 of 248 core eukaryotic genes for fungi were completely identified by evaluating the genome assembly, and 427 of 437 eukaryotic clusters of orthologous groups were identified with an overlap rate > 0.5 when predicted gene sets were assessed (see Supplementary Table S1). These results indicated that our genome assembly and prediction of gene sets for *T. pinophilus* 1–95 were of high quality and confidence.

### Overall genome and proteome comparison

**Figure 2:**
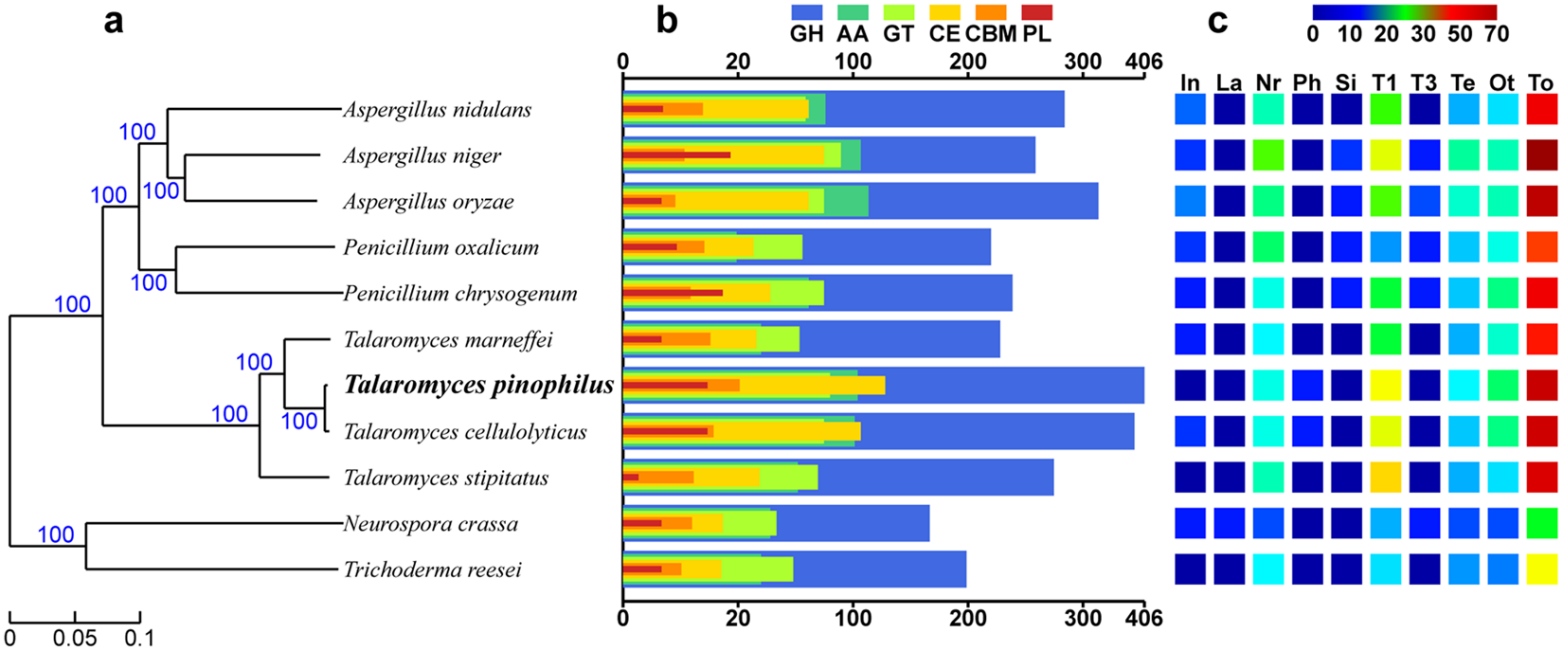
Comparative genomic analysis of *T. pinophilus* and other fungal species. (**a**) Maximum-likelihood phylogenetic tree of *T. pinophilus* and 10 fungal species. (**b**) Comparative analysis of carbohydrate-active enzyme (CAZyme) numbers. GH, glycoside hydrolase; AA, auxiliary activity; GT, glycosyl transferase; CE, carbohydrate esterase; CBM, carbohydrate-binding module; PL, polysaccharide lyase. (**c**) Comparative analysis of secondary metabolite gene cluster numbers. In, Indole and Indole-Nrps; La, Lantipeptide; Nr, Nrps, Nrps-Indole, Nrps-T1pks and Nrps-Terpene; Ph, Phosphonate; Si, Siderophore; T1, T1pks, T1pks-Indole and T1pks-Nrps; T3, T3pks; Te, Terpene-Nrps, Terpene-Nrps-Indole and Terpene-T1pks; Ot, Others; To, total number of secondary metabolite gene clusters. Vertical axes in (**b**) and (**c**) correspond to fungal species in (**a**).

The genome size and number of protein-encoding genes of *T. pinophilus* 1–95 were comparable to other sequenced filamentous fungi (see Supplementary Table S1). A phylogenetic tree constructed based on 2082 single-copy orthologs (see Supplementary Dataset S1) indicated that *T. pinophilus* 1–95 was most closely related to *T. cellulolyticus* Y-94 (Fig. 2a). Analyzing the top hits of a BLASTp (https://blast.ncbi.nlm.nih.gov/Blast.cgi) search of all-vs.-all findings showed that *T. pinophilus* 1–95 and *T. cellulolyticus* Y-94 shared 10,260 orthologous proteins, accounting for 76.16% of the total proteome, with an average amino acid sequence identity of 98.28%. In contrast, low identity was observed to other sequenced *Talaromyces* species^17^, *Trichoderma* sp.^18^, *Penicillium* sp.^19, 20^ and *Aspergillus* sp.^13, 21, 22^, ranging from 70% to 88% (see Supplementary Table S3).

The predicted proteome of *T. pinophilus* 1–95 was 23.84% larger than the proteome of *T. cellulolyticus* Y-94^9^, whereas the genome size of 1–95 was similar to Y-94 (see Supplementary Table S1). The reason for this finding was that the genome sequencing and assembly of *T. pinophilus* 1–95 were of high quality (see Supplementary Table S2), partly due to the lack of gaps in the scaffolds. A comparative analysis of proteins annotated by the GO database between *T. pinophilus* 1–95 and *T. cellulolyticus* Y-94 showed functional differences between the proteins mostly for “biological regulation”, “cellular process” and “metabolic process” in the biological process category and “binding”, “catalytic activity” and “nucleic acid binding transcription factor activity” in the molecular function category (see Supplementary Fig. S2).

We used data from the PE library with an average insert size of 500 bp from *T. pinophilus* 1–95 to map the entire genome sequence of *T. cellulolyticus* Y-94^7^. The PE reads covered 35 scaffolds with 32.06 Mb of sequence from the *T. cellulolyticus* Y-94 genome, resulting in 88% average coverage and 83-times average depth (see Supplementary Table S4). We found 489 genes from *T. cellulolyticus* Y-94 contained insertion-deletion mutations when mapping PE reads from the *T. pinophilus* 1–95 to the genome of *T. cellulolyticus* Y-94; 257 of these occurred in coding sequence regions. Only 33 of the mutated genes showed no similarity with genes in the *T. pinophilus* 1–95 proteome, and approximately half were annotated by the GO database as related to “oxidation-reduction”, “binding” and “catalytic activity”.

### Biomass degrading machinery in *T. pinophilus* 1–95

**Figure 3:**
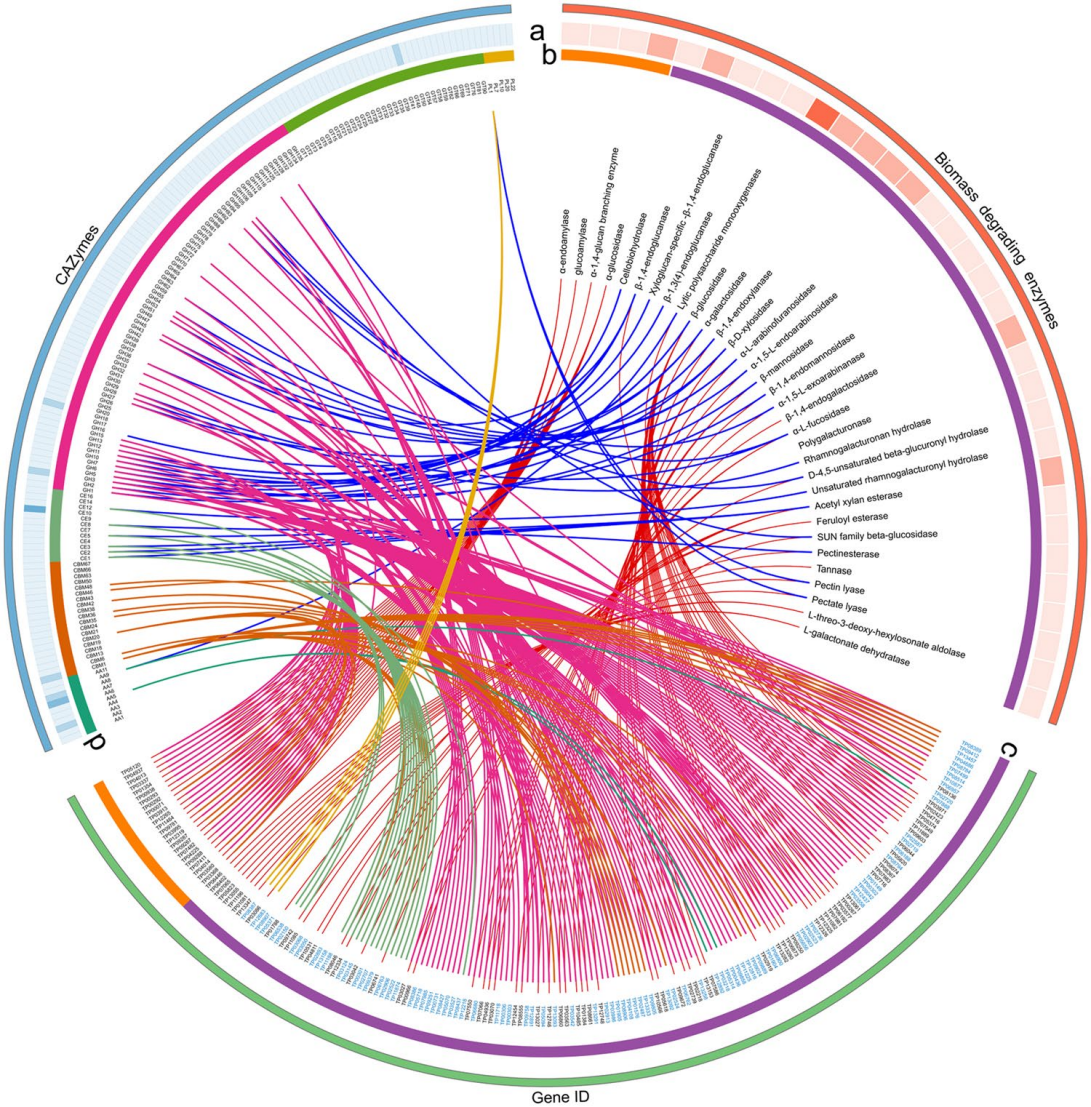
Carbohydrate-active enzyme (CAZymes) genes in *T. pinophilus* 1–95 including plant cell wall-degrading enzymes (CWDEs) and starch-degrading enzymes (SDEs). Blue gene IDs, secreted CWDEs and SDEs. (**a**) Heatmap of different enzyme types. (**b**,**c**) Orange, starch-degrading enzymes; purple, plant CWDEs. (**d**) Colors represent different CAZyme families.

Among the 13,472 unique proteins of *T. pinophilus* 1–95, 803 were annotated as encoding CAZymes using carbohydrate-active enzyme annotations from dbCAN^23^. The putative CAZymes included 72 families of glycoside hydrolases (GHs), 35 families of glycosyl transferases (GTs), 13 families of carbohydrate esterases (CEs), 10 families of auxiliary activities (AAs), 5 families of polysaccharide lyases (PLs) genes and 19 families of carbohydrate-binding modules (CBMs) (Fig. 2b). Further analysis revealed that 156 CAZymes were predicted to be plant cell wall-degrading enzymes (CWDEs), specifically 42 cellulolytic enzymes, 97 hemicellulases and 17 pectinases (Fig. 3; see Supplementary Table S5).

Among the cellulolytic enzymes, 2 cellobiohydrolases (CBHs, EC 3.2.1.91), 8 β-1,4-endoglucanases (EGs, EC 3.2.1.4) and 29 β-glucosidases (BGLs, EC 3.2.1.21) were included as cellulases. Of these, the known cellulases included Cel7A-2 (TP09412), Cel5A (TP03457), Cel5B (TP07499), Cel7B (TP08514) and Bgl3A (TP09042). Compared with known filamentous fungi used for cellulase production, i.e., *T*. *cellulolyticus* Y-94 and *P*. *oxalicum* HP7-1, larger numbers of BGLs were classified into GH families 1 and 3 in the *T. pinophilus* 1–95 genome (see Supplementary Table S5). We examined lytic polysaccharide monooxygenases (LPMO) that catalyze the initial oxidative cleavage of recalcitrant cellulose, resulting in the slow release of oxidized oligosaccharides into solution. LPMO-cellulase synergy is beneficial for degradation of large and highly resistant crystalline cellulose^24, 25^. Only one, TP03971, an ortholog of Cel61A from *P*. *oxalicum* HP7–1, was identified, which was fewer than in *P*. *oxalicum* HP7–1 (see Supplementary Table S5).

The *T. pinophilus* 1–95 genome was rich in hemicellulose-degrading enzymes (97 genes) assigned into 29 predicted CAZyme families; this finding was compared to 77 genes in *T*. *cellulolyticus* Y-94 and 80 genes in *P*. *oxalicum* HP7–1, which includes *Xyn11A* (*TP00436*) and *Xyn10A* (*TP06900*), encoding the important β-1,4-endoxylanases. This result supports the high xylanase activity of *T. pinophilus* 1–95. The predicted hemicellulases in *T*. *pinophilus* 1–95 were divided into 19 types by substrate specificities. The large differences among *T. pinophilus* 1–95, *T*. *cellulolyticus* Y-94 and *P*. *oxalicum* HP7–1 broadly covered most of the listed hemicellulase types (see Supplementary Table S5). For instance, *T. pinophilus* 1–95 possessed more β-D-xylosidases (EC 3.2.1.37), acetyl xylan esterases (EC 3.1.1.72) and feruloyl esterases (EC 3.1.1.73) than the two others. β-D-xylosidases hydrolyze xylobiose or linear xylooligosaccharides to the monomer xylose. Acetyl xylan esterases liberate acetic acid esterifying position 2 and 3 on mono- and di-O-acetylated β-1,4-linked D-xylopyranosyl residues in xylan chains. Feruloyl esterases liberate *trans*-ferulic acid from 5-O-feruloylated L-arabinofuranosyl residues. These enzymes facilitate the hydrolysis of hemicellulose^26^. In contrast, the *T. pinophilus* 1–95 genome possessed a similar number of pectin-degrading enzymes as *T*. *cellulolyticus* Y-94 and *P*. *oxalicum* HP7–1 (see Supplementary Table S5).

*T. pinophilus* 1–95 produces a highly efficient, calcium-independent α-amylase^1^. Application of this enzyme in starch conversion might avoid the drawbacks of calcium ion addition^8^. Among 803 CAZymes, 24 were involved in starch degradation, while 5 to 21 were found in the other 10 investigated filamentous fungi (see Supplementary Table S6). These 24 enzymes were composed of 5 α-amylases (EC 3.2.1.1), which mainly break internal α-1,4-glucosidic linkages and some branched α-1,6-glycosidic linkages from the inner starch chain; 13 α-glucosidases (EC 3.2.1.20), which break α-1,4-linkages in mainly maltose and short maltooligosaccharides to release glucose at nonreducing ends^27^; 5 glucoamylases (EC 3.2.1.3), which mainly hydrolyze α-1,4-glucosidic linkages from the nonreducing ends of starch chains with the release of β-D-glucose^28^; and a 1,4-α-glucan branching enzyme (EC 2.4.1.18) that cleave α-1,4 glucosidic linkages of glucan chain, and then transfer the cut end to 6-position of glucose residue within the cleaved or another glucan chain, resulting the generation of an α-1,6 glucosidic linkage^27, 29^. Comparative analysis indicated that *T*. *pinophilus* 1–95 had more α-glucosidases and glucoamylases than the other investigated fungi (see Supplementary Table S6), supporting that it has a high capacity for starch hydrolysis^1^. Proteomic mass spectrometry and proteome prediction analyses indicated that TP04014 may encode a highly efficient, calcium-independent α-amylase, as reported in Xian *et al*.^1^, but this result needs to be further confirmed experimentally.

### Transcription factor prediction

**Figure 4:**
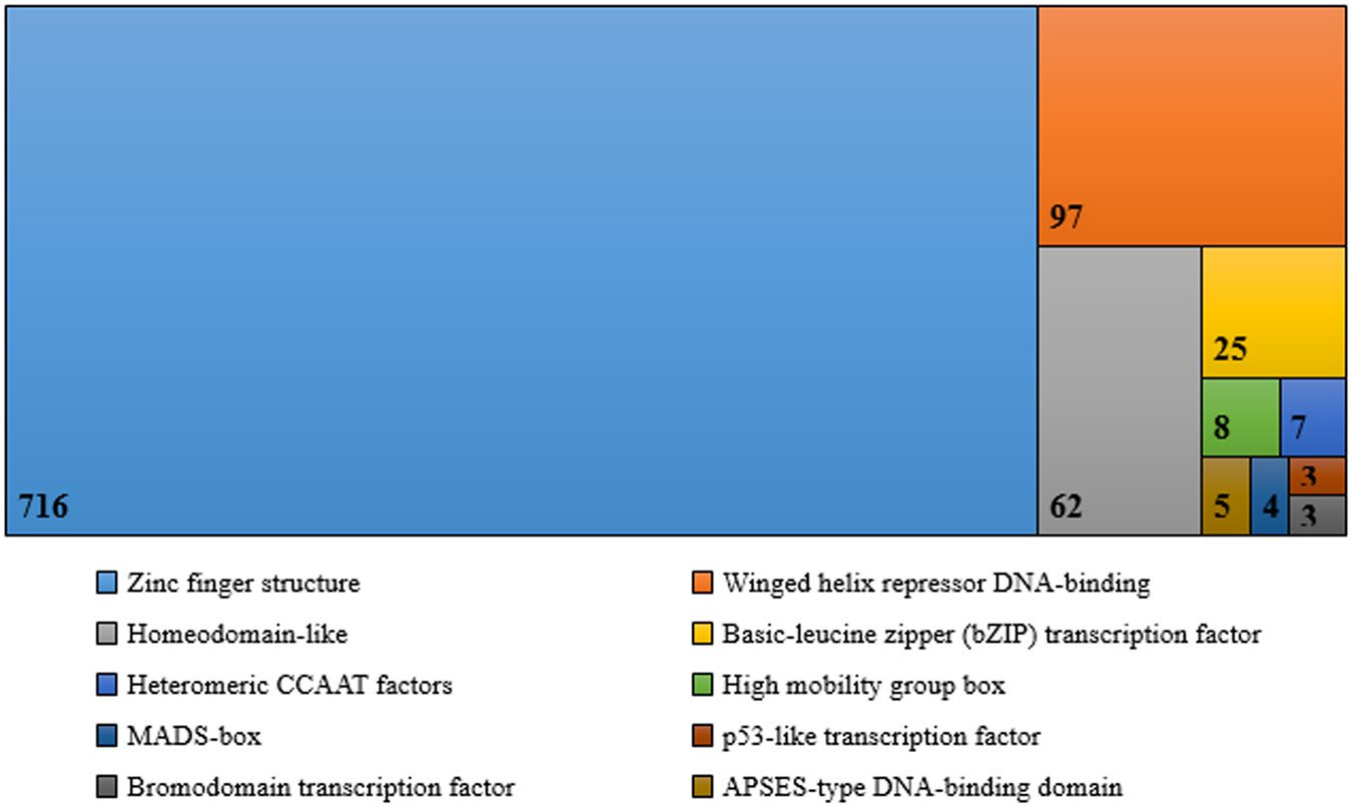
Top 10 types of predicted transcription factors in *T. pinophilus* 1–95.

Transcription factors (TFs) are essential for modulating diverse biological processes by regulating gene expression. In total, 943 TFs were found in the predicted proteome of *T. pinophilus* 1–95. The largest family was proteins (716 members) containing zinc-finger structures, such as the Zn_2_Cys_6_ type, the C2H2 type and CCHC type, followed by the winged helix repressor DNA-binding family (97 members) (Fig. 4). TFs are used in genetic engineering to improve biomass-degrading enzyme yields. We carried out an orthology search of known TFs involved in regulation of lignocellulolytic genes in filamentous fungi against the translated proteins in the *T. pinophilus* 1–95 genome using an identity of 40% and an E-value threshold of 1E-10. Orthologs of most known TFs were identified in *T. pinophilus* 1–95, including the carbon catabolite repressor CreA (TP09972), the cellulase transcription activator CLR-2 (TP10486), the starch degradation regulator AmyR (TP09286) and the xylan degradation regulator XlnR (TP02627) (Table 2). Several related proteins include ACEII, Xpp1, ClrC and BglR, had no ortholog in *T*. *pinophilus* 1–95.

**Table 2:** Orthologs of known transcription factors involved in regulation of biomass-degrading enzyme genes in *T. pinophilus* 1–95.

Protein name	Species	Accession No.	Protein ID in *T*. *pinophilus*
AceA	*Penicillium oxalicum* ^12^	EPS27047.1	TP12581
AceII	*Trichoderma reesei* ^50^	AAK69383.1	Not found
AreA	*Aspergillus nidulans* ^51^	CAA36731	TP08849
AraR	*A. niger* ^52^	A2QJX5.1	TP07534
AmyR	*P. oxalicum* ^53^	EPS29018.1	TP09286
BglR	*T. reesei* ^54^	EGR44729.1	Not found
BrlA	*P. oxalicum* ^55^	EPS25156.1	TP04848
Clr-1	*Neurospora crassa* ^56^	ESA42840	Not found
ClrB	*P. oxalicum* ^53^	EPS31045.1	TP10486
ClrC	*P. oxalicum* ^53^	EPS34061.1	Not found
CreA	*P. oxalicum* ^53^	EPS28222.1	TP09972
Hap2	*P. oxalicum* ^12^	EPS31428.1	TP13257
Hap3	*P. oxalicum* ^12^	EPS27888.1	TP07843
Hap5	*P. oxalicum* ^12^	EPS26080.1	TP12862
PacC	*N. crassa* ^57^	Q7RVQ8.2	Not found
FlbC	*P. oxalicum* ^53^	EPS33410.1	TP08156
Rca1	*N. crassa* ^58^	XP_961398.1	TP08385
Vib1	*N. crassa* ^59^	XP_011394570.1	TP06351
XlnR	*P. oxalicum* ^53^	EPS32714.1	TP02627
Xpp1	*T. reesei* ^60^	EGR46848.1	Not found

### A repertoire of secondary metabolism gene clusters

We found that *T. pinophilus* 1–95 had a wealth of secondary metabolites using the AntiSMASH web service^30^. A total of 68 secondary metabolism gene clusters harboring 401 putative genes were identified. The predicted products of 52 secondary metabolism gene clusters were classified into 8 different types: 28 T1 polyketide synthase (T1PKS) gene clusters, 9 non-ribosomal peptide synthase (NRPS) gene clusters, 9 terpene gene clusters, 2 Nrps-T1pks gene clusters, 1 phosphonate gene cluster, 1 T1pks-Indole gene cluster, 1 T1pks-Nrps gene cluster and 1 Terpene-T1pks gene cluster; the remaining 16 gene clusters synthesized other unknown secondary metabolites (Fig. 2c; see Supplementary Table S7). When compared with known gene clusters for secondary metabolites, eight were predicted to produce emericellin, pestheic acid, azanigerone and azaphilone (data not shown).

### The transporter and secretory system

Transporters are important in microbial growth and reproduction because they assist microbes in the uptake of nutrients and energy from the surrounding environment. In total, 1,238 genes encoding putative transporters belonging to seven superfamilies were identified in the *T. pinophilus* 1–95 genome (see Supplementary Dataset S2). Of these, members of the electrochemical potential-driven transporters (EPTs) were the most abundant, accounting for 54.7%, followed by primary active transporters (18.3%). Among the EPTs, 431 members of a major facilitator superfamily were selected; these are involved in the transport of substances including sugar, drugs and peptides. Of these EPTs, TP13272 and TP06909, respectively, were orthologs of the cellodextrin transporters Cdt-C and Cdt-D, which are crucial for induction of cellulase gene expression by insoluble cellulose^31^.

Many proteins are commonly secreted into extracellular regions, including enzymes involved in biomass degradation. A comprehensive pipeline was designed to predict the *T. pinophilus* 1–95 secretome. *T. pinophilus* 1–95 potentially secreted 1,203 extracellular proteins (8.9% of predicted total proteins), comprising 831 classical and 372 nonclassical secretory proteins (see Supplementary Dataset S3). Using KEGG annotation, 546 putatively secreted proteins were assigned, indicating that these abundant secretory proteins were mainly involved in metabolism, especially carbohydrate metabolism and xenobiotic biodegradation and metabolism (see Supplementary Fig. S3). We found that 323 of these, including 35 nonclassical secreted proteins, were present in the CAZyme database, accounting for 26.8% of the total secretome. The repertoire of secreted CAZymes consisted of 54 GH families, 18 GT families, 12 CE families, 8 AA families, 4 PL families and 13 CBM families. The most common GH family, comprising 188 enzymes, contributed to 58.2% of total secreted CAZymes, followed by the AA families (14.9%). The most prevalent GH CAZyme classes were GH7, GH3, GH5, GH10-13 and GH31, which represent cellulases, endoxylanases and amylases, all of which are required for biomass degradation. The most abundant CBM family was CBM1, accounting for 43.8% of total CBMs, which are known to bind to crystalline cellulose and aid in its enzymatic hydrolysis^32^.

Further analysis indicated that 82 of 323 predicted secretory CAZymes including two nonclassical proteins and six non-CAZymes were identified as CWDEs. Of these, 18 cellulases consisting of two CBHs, seven EGs and nine BGLs were investigated. These cellulases included the major CWDEs for cellulose degradation Cel7A-2 (TP09412), Cel7B (TP08415), Cel5A (TP13457), Cel5B (TP07499), Cel5C (TP08784), Cel45A (TP06957) and Bgl3A (TP09042). We also identified 62 hemicellulose-degrading enzymes and 6 pectin-degrading enzymes, including eight β-1,4-endoxylanases, nine acetyl xylan esterases, four α-galactosidases, 10 α-L-arabinofuranosidases, five α-L-fucosidases, one endo-1,4-β-mannanase and four feruloyl esterases, as well as two pectin esterases, two tannases, one pectate lyase and one pectin lyase (see Supplementary Dataset S3). In addition to CWDEs, 10 starch-degrading enzymes were found in the predicted secretome of *T. pinophilus* 1–95: four α-amylases, one glucoamylase and five α-glucosidases (see Supplementary Dataset S3).

## Discussion

A systematic genetic investigation of filamentous fungi would contribute to genetic engineering of more diverse and productive industrial microbial strains for improving cellulolytic enzyme production. We sequenced, assembled and analyzed the entire *T. pinophilus* 1–95 genome in detail. *T. pinophilus* is a promising filamentous fungus for the industrial production of biomass-degrading enzymes. This study describes the nearly complete genome sequence of a member of the genus *Talaromyces*. The total assembled genome size was 36.51 Mb, which was within the range of filamentous fungi that produce cellulolytic enzymes, including *Penicillium*, *Aspergillus*, *Trichoderma* and *Neurospora* species.

Comparative genome analysis indicated that the most closely related species to *T. pinophilus* 1–95 was *T*. *cellulolyticus* Y-94. *T*. *cellulolyticus* Y-94 was identified as *T*. *pinophilus* based on only an internal transcribed spacer sequence^33^. It was proposed as the new species *T*. *cellulolyticus* in the genus based on morphological and phenotypic differences from *T. pinophilus* ^34^. The reported genome sequence of Y-94 is a draft with a number of gaps^9^. In this study, a nearly complete genome sequence of *T. pinophilus* 1–95 was obtained. The number of protein-encoding genes in *T. pinophilus* was higher than in other investigated fungal strains. This result may be because of the presence of more genes or the result of high-quality SMRT DNA sequencing technology and a different methodology for gene prediction. In particular, a large inventory of CAZymes was found, including CWDEs and starch-degrading enzymes. This result supported the high capacity of this species to degrade biomass, comparable to *T*. *cellulolyticus* Y-94^35^ and *P*. *oxalicum* HP7-1^20^. Of note, *T*. *pinophilus* 1–95 possessed the most BGLs (29 coding genes), glucoamylases (5 coding genes) and α-glucosidases (13 coding genes) among species we compared. BGLs are important for releasing inhibition of cellulase activity^36^. Furthermore, the predicted secretome of *T*. *pinophilus* 1–95 showed that approximately 50% of CWDEs and starch-degrading enzymes were secreted into the extracellular region, including major cellulases, hemicellulases and amylases. This result indicated a promising application of *T. pinophilus* in biorefining. These results also demonstrated that *T*. *pinophilus* 1–95 is more excellent cellular machinery for biomass-degrading enzymes than that of *P. oxalicum* HP7–1 and *T. cellulolyticus* Y-94 as previously observed^20, 35^, meriting further study.

Comparative analysis to 10 filamentous fungi from four genera, *Talaromyces*, *Trichoderma*, *Penicillium* and *Aspergillus*, indicated that *T. pinophilus* 1–95 possessed the most secondary metabolism gene clusters except for *A*. *niger* and *A*. *oryzae*. *T. pinophilus* 1–95 had more T1PKs than *Trichoderma* sp., *Penicillium* sp. and *Aspergillus* sp., and fewer NRPS than *Aspergillus* species. These results suggested that *T. pinophilus* 1–95 has potential for producing bioactive secondary metabolites. Although thus far, several bioactive secondary metabolites have been extracted and characterized from *T. pinophilus* ^6, 7^, according to the genomic data, additional secondary metabolites could be generated using this species.

In summary, this study provided a nearly complete genome sequence for the genus *Talaromyces*. The result provided new insights for a comprehensive understanding of the biomass-degrading enzyme system of *Talaromyces* at the genome level. Detailed comparative genomic analysis revealed a complex biomass-degrading enzyme system in *T*. *pinophilus*, indicating its promising application in biomass biorefineries. This study provides a genome-sequence basis for developing strategies that use *T*. *pinophilus* as a microbial cell factory for production of high-value enzymes and secondary metabolites.

## Materials and Methods

### Culture conditions and genomic DNA extraction

*T. pinophilus* 1–95 was isolated from soil in a dried, ploughed paddy field in Wuzhou, China^1^ and was deposited at the China General Microbiological Culture Collection Center (CGMCC) under accession number CGMCC No. 2645. Total DNA extraction from mycelia was performed using a phenol-chloroform method with some modifications^37^. Mycelia were ground in liquid nitrogen and put in 1 mL lysate reagent (40 mM Tris-HCl, 20 mM sodium acetate, 10 mM ethylenediaminetetraacetic acid, and 1% sodium dodecyl sulfate, pH 8.0) per 100 mg mycelia powder. Genomic DNA was collected by centrifugation at 11,300 × *g* for 10 min.

### Genome sequencing and assembly

The *T. pinophilus* strain 1–95 genome was sequenced using a PacBio RS II platform and Illumina HiSeq 4000 platform at the Beijing Genomics Institute (BGI, Shenzhen, China). Four SMRT cells zero-mode waveguide arrays of sequencing, were used by the PacBio platform to generate the subreads set. PacBio subreads (length < 1 kb) were removed. The program Pbdagcon (https://github.com/PacificBiosciences/pbdagcon) was used for self-correction. Draft genomic unitigs, which are uncontested groups of fragments, were assembled using the Celera Assembler^38^ against a high-quality corrected circular consensus sequence subreads set. Order, distance and orientation of unitigs and combined scaffolds were generated using software SSPACE^39^. An upgraded draft genome was obtained after filling or reducing as many captured gaps as possible using software PBJelly^40^. To improve the accuracy of the genome sequences, GATK (https://www.broadinstitute.org/gatk/) and SOAP tool packages (SOAP2, SOAPsnp, SOAPindel)^41, 42^ were used to make single-base corrections.

A DNA library of 500 bp inserts was constructed and PE sequenced. For generated HiSeq reads, Q20, representing the probability of the incorrectness of the corresponding base call, was detected. If Q20 reads accounted for less than 60%, they were discarded. Software Pilon^43^ used reasonable PE sequence data from Illumina libraries to perform comprehensive variant detection and improve genome assembly.

### Gene detection and functional annotation

Protein-coding genes in the *T. pinophilus* 1–95 genome were predicted independently with the gene prediction programs Augustus (http://bioinf.uni-greifswald.de/augustus/), GeneMark-ES (http://exon.gatech.edu/GeneMark/), Genewise (http://www.ebi.ac.uk/Tools/psa/genewise/), SNAP^14^, and the unsupervised learning system program Glean (https://sourceforge.net/projects/glean-gene/) version 1. Augustus and SNAP, using default parameters, were trained on gene models for *A. oryzae*, *P. oxalicum*, *T. marneffei* and *T. stipitatus* (see Supplementary Table S1). Programs GeneWise and GeneMark-ES were used to obtain different gene sets and worked in a self-training manner. Finally, all prediction gene sets were integrated by Glean.

For functional annotation of translated proteins in the *T. pinophilus* 1–95 genome, a BLASTp version 2.2.28^+^ search against the NCBI NR database (update 05, 2015) and Swiss-Prot and TrEMBL databases (http://www.mrc-lmb.cam.ac.uk/genomes/madanm/pres/swiss2.htm) (update 01, 2016) and KEGG database (http://www.kegg.jp/) version 76, were used to assign general protein function profiles. We used cut-off E-value ≤ 1e-5, overlap 0.4 and identity 30. InterProScan5 (http://www.ebi.ac.uk/interpro/), stand-alone version 55, and GO (http://geneontology.org/) were also used to annotate the predicted proteome. TFs were predicted based on InterPro IDs in the Fungal Transcription Factor Database (http://ftfd.snu.ac.kr/). The hmmsearch program in the HMMER 3.1b2 package (http://hmmer.org/), was used to search all predicted proteomes with the family-specific hidden Markov model profiles of CAZymes from the dbCAN database^26^. Primary results were processed with an E-value threshold of 1E-7. Protein kinases and phosphatases were detected using hmmsearch based on the Eukaryotic Kinase and Phosphatase Database (http://ekpd.biocuckoo.org/). Membrane transport proteins were classified and identified by a BLASTp search against the transport classification database^44^, with E-value threshold 1E-10, overlap 0.4 and identity of 30. AntiSMASH^30^ was used to annotate secondary metabolism gene clusters.

### Phylogenetic analysis

An all-against-all pairwise BLASTp similarity search was performed using proteomes from 11 filamentous fungi (see Supplementary Table S2) with E-value cutoff 1E-7, according to the method described previously^45^. We selected 2,082 single-copy genes from 118,099 genes in 11 fungal genomes. MUSCLE (http://www.ebi.ac.uk/Tools/msa/muscle/) version 3.7 with default parameters was used to perform multiple sequence alignment of single-copy genes. A neighbor-joining tree was calculated using TreeBeST^46^ with bootstrapping set to 100. The phylogenetic tree was visualized using SVGKit (http://svgkit.sourceforge.net/) and PERL scripts.

### Secretome prediction

The total set of 13,472 proteins of *T. pinophilus* strain 1–95 was analyzed using Secretome P^47^ v1.0 and SignalP^48^ v4.0 for *ab initio* prediction of classical and nonclassical secretory proteins, except for 11 proteins with more than 4,000 amino acid residues. All proteins containing signal peptides were selected, with proteins without signal peptides chosen as candidates in cases of neural network score ≥ 0.6. All selected proteins were analyzed by TargetP^49^ v1.1 with localization = secretory pathway and RC≤ 4 as screening criteria. Protein sets were scrutinized for the presence of transmembrane domains using TMHMM v2.0 (http://www.cbs.dtu.dk/services/TMHMM/) and for glycosylphosphatidyl inositol anchors use web server PredGPI (http://gpcr.biocomp.unibo.it/predgpi/). Software tRNAscan-SE (http://lowelab.ucsc.edu/tRNAscan-SE/) version 1.3 was used for transfer RNA prediction using the *T. pinophilus* 1–95 genome with option C and other default parameters.

## Electronic supplementary material

Supplementary Infromation for SREP-16-39329

Dataset 1

Dataset 2

Dataset 3
