# Supplementary material for: Genome sequencing and analysis of Talaromyces pinophilus provide insights into biotechnological applications
Source: Sci Rep. 2017 Mar 28;7:490. doi: 10.1038/s41598-017-00567-0 (PMC5428652; doi:10.1038/s41598-017-00567-0)
Supplement: Supplementary file 1 — Supplementary Infromation for SREP-16-39329 [file 41598_2017_567_MOESM1_ESM.pdf]

Supplementary Information

Genome sequencing and analysis of *Talaromyces pinophilus* provide insights into biotechnological applications

Cheng-Xi Li†, Shuai Zhao†, Ting Zhang, Liang Xian, Lu-Sheng Liao, Jun-Liang Liu, Jia-Xun Feng*

State Key Laboratory for Conservation and Utilization of Subtropical Agro-bioresources, College of Life Science and Technology, Guangxi University, 100 Daxue Road, Nanning, Guangxi 530004, People's Republic of China.

*Corresponding author. E-mail address: jiaxunfeng@sohu.com (J.-X. Feng)

†These authors contributed equally to this work.

Supplementary files provided with this submission:

Figure S1. Venn diagram showing the unique and shared proteins in *T. pinophilus* 1-95 annotated using the Non-redundant (NR), UniProt, Kyoto Encyclopaedia of Genes and Genomes (KEGG), the Clusters of Orthologous Groups of proteins (COG) and Gene Ontology (GO) databases.

Figure S2. Functional annotation of proteins as determined using the Gene Ontology (GO) database, and comparative analysis of the identified proteins in *T. pinophilus* 1-95 and *T. cellulolyticus* Y-94.

Figure S3. Functional annotation of the secreted proteins of *T. pinophilus* 1-95 using the Kyoto Encyclopaedia of Genes and Genomes (KEGG) database.

Table S1. Comparison analysis of genome features among *T. pinophilus* 1-95 and another 10 filamentous fungi producing biomass degrading enzymes.

Table S2. Assessment analysis of *T. pinophilus* 1-95 genome by software CEGMA and BUSCO.

Table S3. Comparative analysis of homologous proteins among *T. pinophilus* 1-95 and another 10 filamentous fungi producing biomass degrading enzymes.

Table S4. Paired-end (PE) libraries with average insert sizes 500 bp mapped the whole genome sequence of *Talaromyces cellulolyticus* Y-94.

Table S5. Comparison of plant cell wall degrading enzymes (CWDEs) among *T. pinophilus* 1-95, *T. cellulolyticus* Y-94 and *Penicillium oxalicum* HP7-1.

Table S6. Comparison of starch degrading enzymes among *T. pinophilus* 1-95 and another 10 filamentous fungi producing biomass degrading enzymes.

Table S7. The predicted secondary metabolism gene clusters in *T. pinophilus* 1-95 and the comparison analysis among with another 10 filamentous fungi producing biomass degrading enzymes.

Dataset S1. Orthologs used for the construction of phylogenetic tree among *Talaromyces pinophilus* 1-95 and another 10 filamentous fungi producing biomass degrading enzymes.

Dataset S2. The putative transporters in *Talaromyces pinophilus* 1-95.

Dataset S3. The secretome of *Talaromyces pinophilus* 1-95 and function annotation

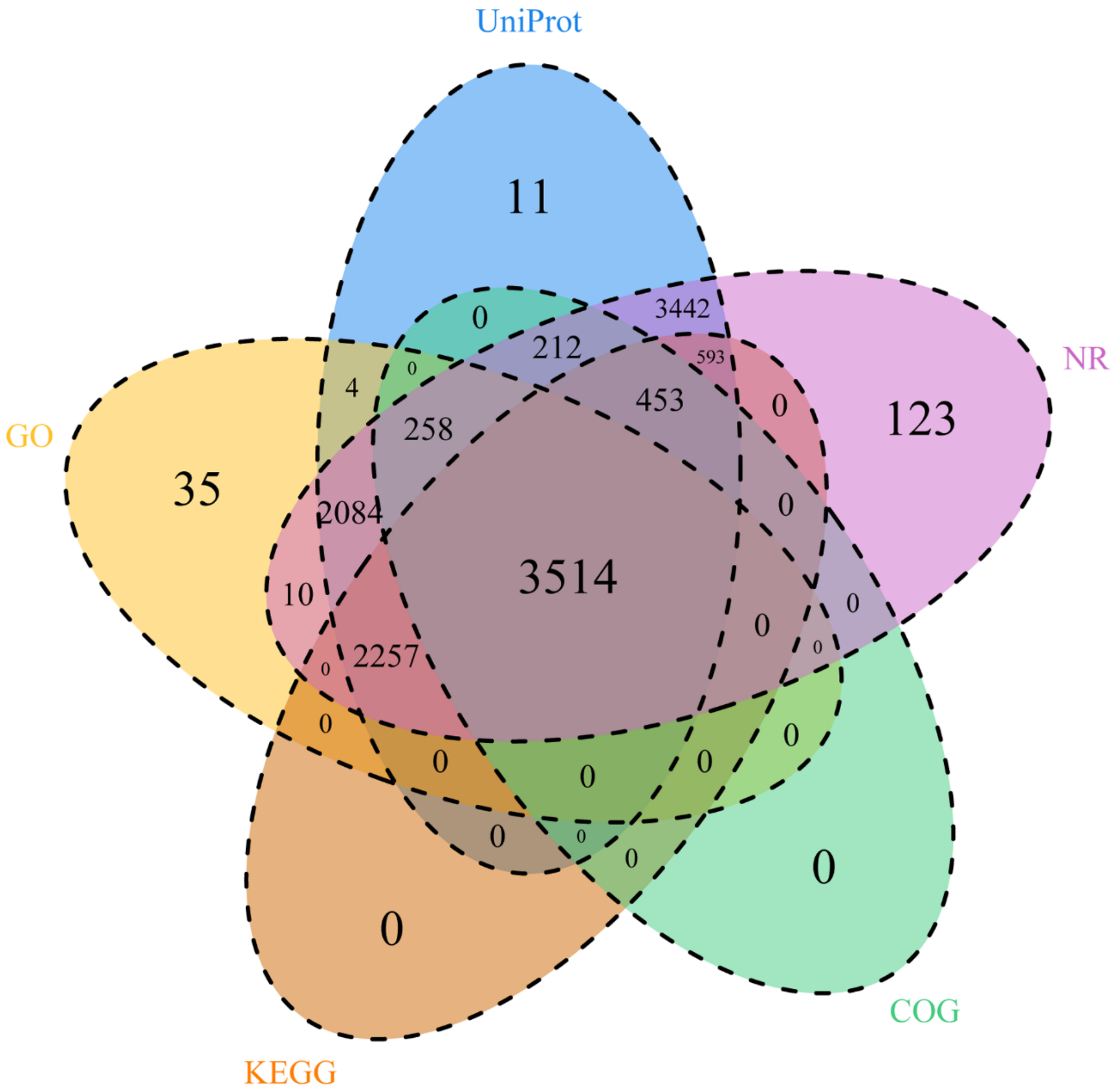

Supplementary Figure S1. Venn diagram showing the unique and shared proteins in *T. pinophilus* 1-95 annotated using the Non-redundant (NR), UniProt, Kyoto Encyclopaedia of Genes and Genomes (KEGG), the Clusters of Orthologous Groups of proteins (COG) and Gene Ontology (GO) databases.

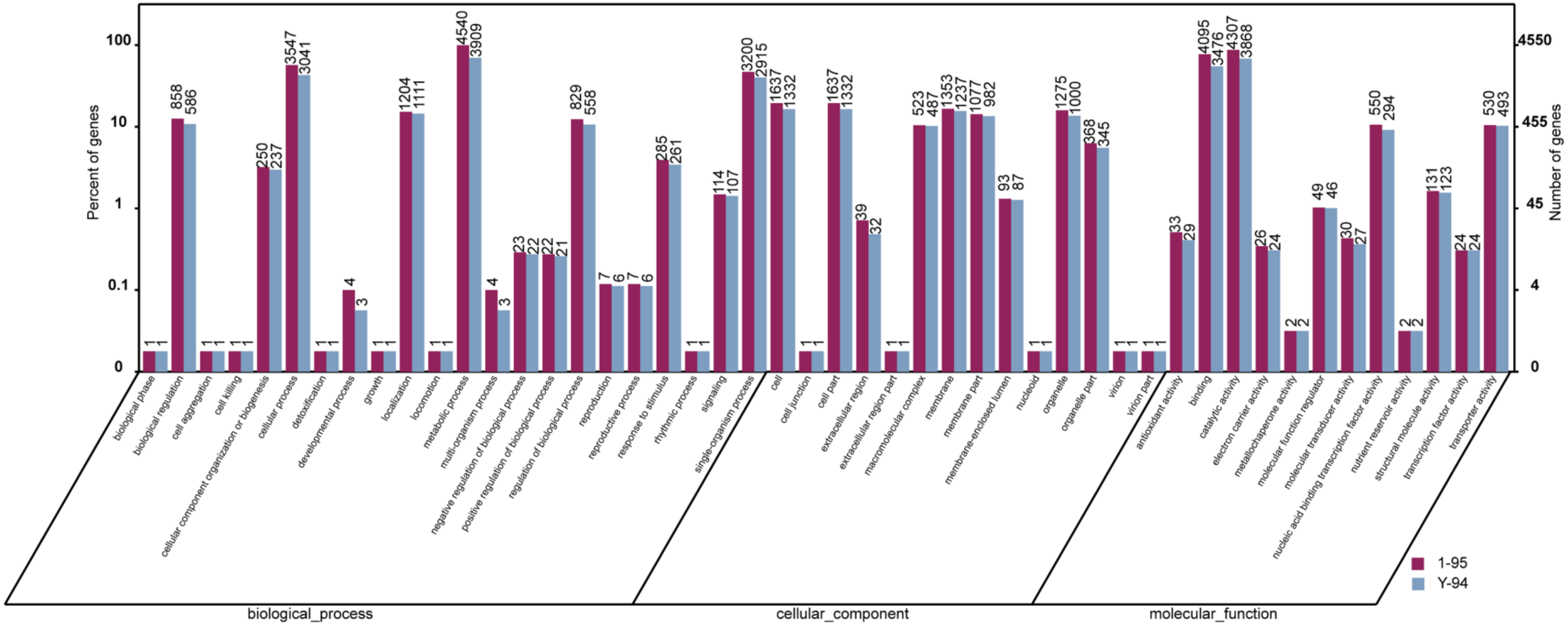

Supplementary Figure S2. Functional annotation of proteins as determined using the Gene Ontology (GO) database, and comparative analysis of the identified proteins in *T. pinophilus* 1-95 and *T. cellulolyticus* Y-94.

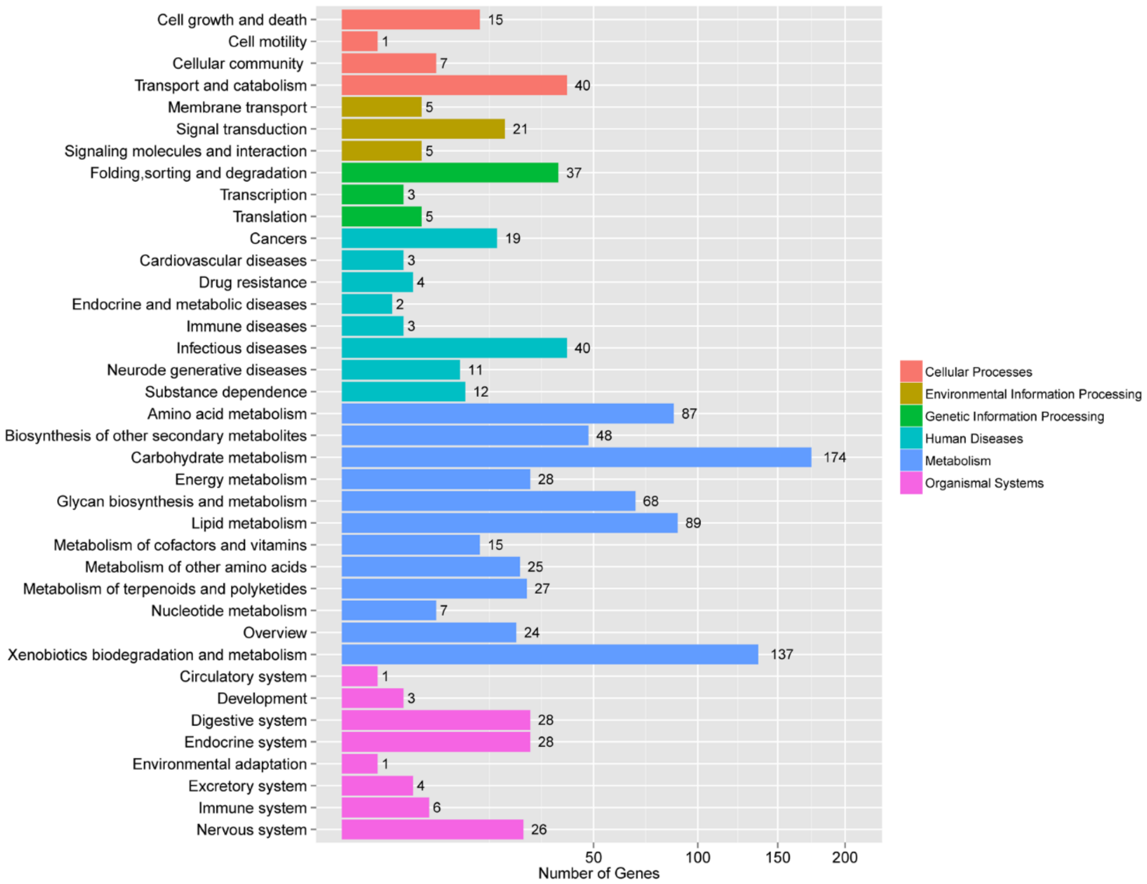

Supplementary Figure S3. Functional annotation of the secreted proteins of *T. pinophilus* 1-95 using the Kyoto Encyclopaedia of Genes and Genomes (KEGG) database.

Supplementary Table S1. Comparison analysis of genome features among *T. pinophilus* 1-95 and another 10 filamentous fungi producing biomass degrading enzymes.

Strains	Genome size (Mb)	Protein Number*	GC (%)	Data Resource
T. pinophilu 1-95	36.51	13472	50.60	This study
Aspergillus nidulans FGSC A4	30.24	9541	50.32	GCF_000149205.1
A. oryzae 3.042	36.57	11321	48.32	GCA_000269785.2
A. niger CBS 513.88	34.00	10503	50.35	GCF_000002855.3
Penicillium chrysogenum P2niaD18	32.52	11090	48.95	GCA_000710275.1
P. oxalicum HP7-1	30.65	9834	50.65	GCA_001723175.1
T. marneffei ATCC 18224	28.64	10002	46.67	GCF_000001985.1
T. stipitatus ATCC 10500	35.68	12442	46.07	GCF_000003125.1
T. cellulolyticus Y-94	36.40	10867	46.63	GCA_000829775.1
Trichoderma reesei QM6a	33.9	9120	52.82	GCF_000167675.1
Neurospora crassa OR74A	41.10	9,907	48.23	GCF_000182925.2

*indicates that the number of genes is obtained through Perl script parsing GBK format.

Supplementary Table S2. Assessment analysis of *T. pinophilus* 1-95 genome by software CEGMA and BUSCO.

**Strain**	**CEGMA**					**BUSCO**					
	Genome assembly (248 genes)			Gene Set (437 genes)		Genome Assembly (1438 genes)			Gene Set (1438 genes)		
	Complete matching genes	Partial matching genes	Matching percent (%)	Overlap genes	Percent (%)	Complete matching genes	Partial matching genes	Matching percent (%)	Complete matching genes	Partial matching genes	Matching percent (%)
T. pinophilus 1-95	238	5	97.98	427	97.71	1419	15	98.67	1431	6	99.51
T. cellulolyticus Y-94	233	7	96.77	383	88.45	1411	18	98.12	1383	39	96.17

Supplementary Table S3. Comparative analysis of homologous proteins among *T. pinophilus* 1-95 and another 10 filamentous fungi producing biomass degrading enzymes.

**Species**	***Aspergillus nidulans* FGSC A4**	***A. niger* CBS 513.88**	***A. oryzae* 3.042**	***Neurospora crassa* OR74a**	***Penicillium chrysogenum* P2niaD18**	***P. oxalicum* HP7-1**	***T. marneffei* ATCC 18224**	***T. stipitatus* ATCC 10500**	***T. cellulolyticus* Y-94**	***Trichoderma reesei* QM6a**
Number of homologous proteins	3119	3824	3599	1407	3378	3520	8342	8098	10260	1626
Average identity (%)	72.7	73	72.7	71.33	72.66	72.43	88.23	84.29	98.28	70.922

Supplementary Table S4. Paired-end (PE) libraries with average insert sizes 500 bp mapped the whole genome sequence of *Talaromyces cellulolyticus* Y-94.

ChrID	Reference size (bp)	Covered length (bp)	Coverage (%)	Depth
DF933797	16189	132	0.82	9.5
DF933798	7171	0	0	0
DF933799	249371	233300	93.56	91
DF933800	543574	476104	87.59	78
DF933801	7505	0	0	0
DF933802	3566	0	0	0
DF933803	14379	134	0.93	85
DF933804	4507	0	0	0
DF933805	5272	4854	92.07	500
DF933806	8137	0	0	0
DF933807	726475	625158	86.05	83
DF933808	9682	0	0	0
DF933809	1090089	955490	87.65	83
DF933810	23213	0	0	0
DF933811	3494755	3243615	92.81	84
DF933812	52934	141	0.27	26
DF933813	2643372	2503190	94.7	85
DF933814	2887201	2697584	93.43	86
DF933815	3210	0	0	0
DF933816	4047	125	3.09	1
DF933817	9580	0	0	0
DF933818	1336228	1156154	86.52	86
DF933819	571201	510571	89.39	88
DF933820	1071518	994881	92.85	84
DF933821	3550	0	0	0
DF933822	22512	8184	36.35	91
DF933823	3934	0	0	0
DF933824	31772	4145	13.05	310
DF933825	46236	318	0.69	1.6
DF933826	15853	141	0.89	11
DF933827	2017	0	0	0
DF933828	2333	2260	96.87	73
DF933829	3612999	3170374	87.75	84
DF933830	4195530	3952279	94.2	86
DF933831	4609	0	0	0
DF933832	4347	0	0	0
DF933833	2041	2035	99.71	70
DF933834	826585	749013	90.62	80

DF933835	1358518	1166859	85.89	83
DF933836	2056	0	0	0
DF933837	1279461	1122105	87.7	84
DF933838	2486755	2269975	91.28	86
DF933839	969434	819449	84.53	84
DF933840	3189086	2579602	80.89	81
DF933841	5308	0	0	0
DF933842	17731	0	0	0
DF933843	844840	668455	79.12	84
DF933844	9262	243	2.62	12
DF933845	7524	0	0	0
DF933846	577942	480141	83.08	81
DF933847	42575	0	0	0
DF933848	10449	0	0	0
DF933849	6082	0	0	0
DF933850	10834	0	0	0
DF933851	18859	0	0	0
DF933852	61417	161	0.26	4.7
DF933853	9072	0	0	0
DF933854	20907	274	1.31	190
DF933855	3406	0	0	0
DF933856	1913789	1661084	86.8	80
**Total**	**36402801**	**32058530**	**88.07**	**83**

Supplementary Table S5. Comparison of plant cell wall degrading enzymes (CWDEs) among *T. pinophilus* 1-95, *T. cellulolyticus* Y-94 and *Penicillium oxalicum* HP7-1.

Enzyme	EC Number	CAZy Family	*T. pinophilus* 1-95	*T. cellulolyticus* Y-94	*P. oxalicum* HP7-1
**1. Cellulose-degrading enzymes**					
Cellobiohydrolase	3.2.1.91	GH6	TP08389		POX04786
		GH7	TP09412	TCE0_004r00180	POX02490
					POX05587
β-1,4-endoglucanase	3.2.1.4	GH5	TP13457	TCE0_013f01292	POX01166
			TP04686	TCE0_017r03786	POX01896
			TP08784	TCE0_024f07747	POX02740
			TP07499	TCE0_033r09253	POX04137
					POX06147
					POX01206
		GH7	TP08514	TCE0_024r07350	POX05571
		GH12	TP10877	TCE0_042r14939	POX06983
					POX07535
		GH45	TP06957	TCE0_023r07056	POX05570
			TP08136	TCE0_033r08342	
Xyloglucan-specific β-1,4-endoglucanase	3.2.1.151	GH12	TP02720	TCE0_015f02847	POX00963
β-1,3(4)-endoglucanase	3.2.1.6	GH16	TP07888	TCE0_033r08690	POX06959
					POX00652
Lytic polysaccharide monooxygenases	（LPMOs）	AA9	TP03971	TCE0_044r17407	POX02308
					POX05968
					POX08897

					POX09256
β-glucosidase	3.2.1.21	GH1	TP02423	TCE0_015r02411	POX01473
			TP04716	TCE0_017r03738	POX03062
			TP05374	TCE0_018f05835	POX06079
			TP07549	TCE0_033f09178	POX08882
			TP11889	TCE0_060r18631	
		GH3	TP09603	TCE0_011f00448	POX00968
			TP02587	TCE0_015f02654	POX01646
			TP02719	TCE0_015r02845	POX03641
			TP06044	TCE0_018f04835	POX04358
			TP06188	TCE0_018r04615	POX06835
			TP05820	TCE0_018r05172	POX07963
			TP08794	TCE0_024f07765	POX08491
			TP08074	TCE0_033f08430	POX08984
			TP08367	TCE0_033r08027	
			TP07983	TCE0_033r08553	
			TP07716	TCE0_033r08939	
			TP01149	TCE0_034f10525	
			TP00302	TCE0_034f11821	
			TP09042	TCE0_039f12945	
			TP12437	TCE0_041f14010	
			TP03558	TCE0_044f16774	
			TP13061	TCE0_050r18181	
			TP00267	TCE0_004r00287	
			TP03577	TCE0_038f12659	
			TP06192		

			TP07981		
			TP11082		
			TP12325		
			TP12326		
**2. Hemicellulose-degrading enzymes**					
α-galactosidase	3.2.1.22	GH27	TP02736	TCE0_015f02871	POX04444
			TP02523	TCE0_015r02550	POX09085
			TP02903	TCE0_015r03131	
			TP05656	TCE0_018f05406	
		GH36	TP09250	TCE0_039f13249	
			TP06873	TCE0_047f17698	
			TP13280	TCE0_013f01052	
			TP13282		
β-1,4-endoxylanase	3.2.1.8	GH10	TP06900	TCE0_023r06982	POX05916
					POX08990
					POX00063
		GH11	TP01904	TCE0_015f01698	POX01417
			TP02919	TCE0_015r03151	POX02723
			TP09889	TCE0_022f06941	POX03430
			TP09024	TCE0_039r12923	POX06783
			TP12816	TCE0_041r13448	POX08484
			TP11225	TCE0_042f15439	
			TP09858		
			TP00436		
		GH30			POX04274
					POX06601
β-D-xylosidase	3.2.1.37	GH3	TP05314	TCE0_018r05917	POX00007

			TP03218	TCE0_044f16290	POX06571
			TP12892	TCE0_050r18425	
			TP07586		
		GH43	TP13153	TCE0_013r00881	POX04995
			TP13258	TCE0_013r01023	POX08661
			TP02218	TCE0_015r02141	POX00006
			TP02739	TCE0_015r02874	POX01744
			TP06762	TCE0_047f17875	
			TP09672		
α-L-arabinofuranosidase	3.2.1.55	GH43	TP02524	TCE0_015r02553	POX05240
					POX06600
					POX07665
					POX07891
		GH51	TP13092	TCE0_013f00789	POX04752
			TP05618	TCE0_018r05464	POX06355
			TP10566	TCE0_042f14472	POX07441
		GH54	TP09805	TCE0_011f00699	POX01914
			TP13333	TCE0_013f01127	
			TP02487	TCE0_015r02500	
			TP01576	TCE0_034f09917	
			TP04108	TCE0_044r17624	
		GH62	TP08906	TCE0_039f12752	POX05540
			TP01905	TCE0_015r01700	POX06599
			TP03986	TCE0_044f17429	
α-1,5-L-endoarabinosidase	3.2.1.99	GH43	TP02913		POX00923
			TP12748		POX01921
			TP13291	TCE0_013f01067	POX08140

					POX09154
β-mannosidase	3.2.1.25	GH2	TP08661	TCE0_024f07565	POX00759
			TP01384	TCE0_034f10187	POX01961
			TP10485	TCE0_042f14333	
			TP03542	TCE0_044f16757	
			TP03563	TCE0_044r16782	
			TP06860	TCE0_047f17717	
β-1,4-endomannanase	3.2.1.78	GH5	TP13093	TCE0_013f00791	POX01937
			TP12746		POX07285
		GH26			POX07536
α-1,5-L-exoarabinanase	3.2.1.-	GH93	TP05094	TCE0_017r03210	POX00008
			TP13027	TCE0_050f18245	POX01274
			TP12891	TCE0_050r18426	POX03420
			TP09758		
β-1,4-endogalactosidase	3.2.1.89	GH53	TP12454	TCE0_041r13985	POX06689
α-glucosiduronase	3.2.1.139	GH67			POX04275
α-L-fucosidase	3.2.1.51	GH29	TP00303	TCE0_034f11818	
			TP00306	TCE0_034r11813	
			TP11718	TCE0_060f18889	
			TP03070		
		GH95	TP04936	TCE0_017r03422	POX03063
			TP07066	TCE0_023r07212	
			TP06680	TCE0_047r18022	
			TP07550		
			TP12218		
Polygalacturonase	3.2.1.15	GH28	TP08437	TCE0_033f07936	POX03447
			TP03527	TCE0_044f16730	POX03730

			TP05070		POX04815
					POX07304
					POX04570
					POX06790
					POX08286
					POX05580
Rhamnogalacturonan hydrolase	3.2.1.171	GH28	TP08427	TCE0_033r07948	POX02024
			TP00731	TCE0_034f11163	POX04377
			TP09251	TCE0_039f13250	POX01225
			TP07985		
D-4,5-unsaturated β-glucuronyl hydrolase	3.2.1.-	GH88			POX06998
Unsaturated rhamnogalacturonyl hydrolase	3.2.1.172	GH105	TP05718	TCE0_018r05318	POX08875
			TP08386	TCE0_033f08000	
			TP00966	TCE0_034f10811	
			TP03027	TCE0_044r16075	
Rhamnogalacturonan acetylesterase	3.1.1.86	CE12	TP11874	TCE0_060f18653	POX08476
					POX07373
Acetyl xylan esterase	3.1.1.72	CE1	TP02522	TCE0_015f02549	POX01218
			TP02906	TCE0_015r03136	
		CE2	TP06763	TCE0_047r17873	POX08861
			TP06741	TCE0_047r17910	
		CE3	TP09379	TCE0_004r00149	
			TP02707	TCE0_015r02825	
			TP00301	TCE0_034r11823	
			TP03042	TCE0_043r15518	
			TP03145	TCE0_044r16257	
			TP03124		

			TP12334		
		CE5	TP08046	TCE0_033f08462	POX03711
			TP11188		
Feruloyl esterase	3.1.1.73	CE1			POX00197
					POX09137
		Not assigned	TP13158	TCE0_013r00888	POX00727
			TP02893	TCE0_015r03115	POX01117
			TP04811	TCE0_017r03600	POX00668
			TP10531	TCE0_042f14421	
			TP03050		
			TP03068		
			TP11085		
SUN family beta-glucosidase	3.2.1.-	GH132	TP08555	TCE0_024f07410	POX01158
					POX06380
**3. Pectin-degrading enzymes**					
Pectinesterase	3.1.1.11	CE8	TP09742	TCE0_011f00620	POX07932
			TP02150	TCE0_015f02043	POX08067
			TP06538	TCE0_038r12590	POX00760
Tannase	3.1.1.20	Not assigned	TP01788	TCE0_015r01535	POX00729
			TP05371	TCE0_018r05839	POX08434
			TP08907	TCE0_039r12754	
Pectin lyase	4.2.2.10	PL1	TP12683	TCE0_041f13655	POX04920
			TP03086	TCE0_044r16137	POX09802
Pectate lyase	4.2.2.2	PL1	TP08387	TCE0_033f07999	POX07890
			TP13347	TCE0_013f01148	
Rhamnogalacturonan endolyase	4.2.2.23	PL4			POX05310

					POX06611
					POX08785
L-threo-3-deoxy-hexylosonate aldolase	4.1.2.54	Not assigned	TP01081	TCE0_034f10618	POX02774
			TP11196	TCE0_042r15394	POX07471
			TP13055	TCE0_050f18191	
L-galactonate dehydratase	4.2.1.146	Not assigned	TP05823	TCE0_018f05169	POX04114
			TP07065	TCE0_023f07210	
			TP06402	TCE0_038r12406	
			TP06446	TCE0_038r12473	

Supplementary Table S6. Comparison of starch degrading enzymes among *T. pinophilus* 1-95 and another 10 filamentous fungi producing biomass degrading enzymes.

Protein Name	EC Number	***T. pinophilus* 1-95**	***Aspergillus nidulans* FGSC A4**	***A. niger* CBS 513.88**	***A. oryzae* 3.042**	***Neurospora crassa* OR74A**	***T. cellulolyticus* Y-94**	***T. marneffei* ATCC 18224**	***T. stipitatus* ATCC 10500**	***Trichoderma reesei* QM6a**	***Penicillium chrysogenum* P2niaD18**	***P. oxalicum* HP7-1**
α-amylase	3.2.1.1	TP03368	AN2018.2	ANI_1_1100184	Ao3042_01517	NCU05873	TCE0_033r09409	PMAA_058260	TSTA_013540	TRIREDRAFT_105956	EN45_014170	POX03230
		TP03580	AN3308.2	ANI_1_1284084	Ao3042_01518	NCU08131	TCE0_039f13307	PMAA_063250	TSTA_044280		EN45_045100	POX03231
		TP04014	AN3309.2	ANI_1_1524104	Ao3042_03622	NCU09486	TCE0_044f16809	PMAA_064990	TSTA_082630		EN45_073300	POX09352
		TP07411	AN3388.2	ANI_1_1810134	Ao3042_05212	NCU09805	TCE0_044f17478	PMAA_096850	TSTA_084740		EN45_090540	
		TP09288	AN3402.2	ANI_1_260044			TCE0_044r16495		TSTA_089790		EN45_090550	
			AN4507.2	ANI_1_3270014							EN45_105210	
			AN6324.2	ANI_1_362084							EN45_107900	
				ANI_1_460094							EN45_107910	
				ANI_1_820104								
Glucoamylase	3.2.1.3	TP04225	AN7402.2	ANI_1_820034	Ao3042_08133	NCU01517	TCE0_039r13271	PMAA_096710	TSTA_044290	TRIREDRAFT_1885	EN45_015780	POX01356
		TP07482	AN8904.2		Ao3042_10648		TCE0_039r13304	PMAA_096840	TSTA_044610		EN45_097990	POX02412
		TP09267					TCE0_043f15539		TSTA_106690		EN45_105200	POX04642
		TP09287										
		TP12319										
1,4-α-glucan branching enzyme	2.4.1.18	TP03995	AN2314.2	ANI_1_602124	Ao3042_08360	NCU05429	TCE0_044f17444	PMAA_068370	TSTA_088790	TRIREDRAFT_123368	EN45_072830	POX04938
α-glucosidase	3.2.1.20	TP09781	AN0941.2	ANI_1_1098184	Ao3042_05838	NCU02583	TCE0_011r00674	PMAA_002150	TSTA_023890	TRIREDRAFT_59578	EN45_014160	POX03452
		TP11464	AN2017.2	ANI_1_1476014	Ao3042_06557	NCU04674	TCE0_018f06215	PMAA_007280	TSTA_041120	TRIREDRAFT_82235	EN45_050490	POX03741
		TP12265	AN3516.2	ANI_1_1804024	Ao3042_07614	NCU06523	TCE0_034f12152	PMAA_015950	TSTA_041820		EN45_053200	POX03889
		TP03913	AN4843.2	ANI_1_850114	Ao3042_07671	NCU07860	TCE0_034r10854	PMAA_099560	TSTA_101210		EN45_075930	POX06242
		TP00071	AN7345.2		Ao3042_10501	NCU09281	TCE0_043r15609		TSTA_101220		EN45_084270	POX06751
		TP05786	AN8953.2		Ao3042_10503		TCE0_044r16449		TSTA_106040		EN45_084670	

		TP00293			Ao3042_11801		TCE0_044r17312		TSTA_106170		EN45_086140	
		TP00938					TCE0_060r19238		TSTA_112550		EN45_091760	
		TP01354									EN45_097770	
		TP03337										
		TP04013										
		TP04937										
		TP05120										

Supplementary Table S7. The predicted secondary metabolism gene clusters in *T. pinophilus* 1-95 and the comparison analysis among with another 10 filamentous fungi producing biomass degrading enzymes.

**Cluster**	***T. pinophilus* 1-95**	***T. cellulolyticus* Y-94**	***Penicillium oxalicum* HP7-1**	***Neurospora crassa* OR74A**	***T. marneffei* ATCC 18224**	***T. stipitatus* ATCC 10500**	***Trichoderma reesei* QM6a**	***Aspergillus nidulans* FGSC A4**	***A. niger* CBS 513.88**	***A. oryzae* 3.042**	***P. chrysogenum* P2niaD18**
Indole	0	2	2	1	0	0	0	4	2	5	0
Indole-Nrps	0	0	0	0	1	0	0	0	0	0	1
Lantipeptide	0	0	0	1	0	0	0	0	0	0	0
Nrps	9	9	12	3	7	9	6	10	15	12	8
Nrps-Indole	0	0	1	0	0	0	0	1	0	1	0
Nrps-T1pks	2	2	3	0	3	3	4	2	8	2	3
Nrps-Terpene	0	0	0	0	0	1	0	0	0	0	0
Other	16	15	11	3	12	9	5	9	13	13	15
Phosphonate	1	1	0	0	0	0	0	0	0	0	0
Siderophore	0	0	1	0	0	0	0	0	2	1	1
T1pks	28	28	5	7	16	30	9	22	27	22	18
T1pks-Indole	1	1	0	0	1	0	0	0	0	1	0
T1pks-Nrps	1	0	1	0	1	3	0	0	2	0	0
T3pks	0	0	1	1	0	0	0	0	1	3	1
Terpene	9	7	7	3	7	7	6	7	14	11	7
Terpene-Nrps	0	0	0	0	0	0	0	0	0	1	1
Terpene-Nrps-Indole	0	0	1	0	0	0	0	0	0	0	0
Terpene-T1pks	1	1	0	0	0	0	0	0	0	0	0
**Total**	**68**	**66**	**45**	**19**	**48**	**62**	**30**	**55**	**84**	**72**	**55**
